# Myopia progression risk assessment score (MPRAS): a promising new tool for risk stratification

**DOI:** 10.1038/s41598-023-35696-2

**Published:** 2023-05-31

**Authors:** Manoj K. Manoharan, Swapnil Thakur, Rohit Dhakal, Satish K. Gupta, Jacinth J. Priscilla, Shashank K. Bhandary, Alok Srivastava, Srinivas Marmamula, Nitish Poigal, Pavan K. Verkicharla

**Affiliations:** 1 Myopia Research Lab, Prof. Brien Holden Eye Research Centre, Brien Holden Institute of Optometry and Vision Sciences, L V Prasad Eye Institute, Kallam Anji Reddy Campus, Hyderabad, Telangana 500034 India; 2 Infor Myopia Centre, L V Prasad Eye Institute, Hyderabad, Telangana India; 3 L V Prasad Eye Institute, Hyderabad, Telangana India; 4 Sri Innovation and Research Foundation, Ghaziabad, Uttar Pradesh India; 5 Allen Foster Community Eye Health Research Centre, Gullapalli Pratibha Rao International Centre for Advancement of Rural Eye Care, L V Prasad Eye Institute, Hyderabad, Telangana India

**Keywords:** Risk factors, Paediatric research, Translational research

## Abstract

Timely identification of individuals “at-risk” for myopia progression is the leading requisite for myopia practice as it aids in the decision of appropriate management. This study aimed to develop ‘myopia progression risk assessment score’ (MPRAS) based on multiple risk factors (10) to determine whether a myope is “at-risk” or “low-risk” for myopia progression. Two risk-score models (model-1: non-weightage, model-2: weightage) were developed. Ability of MPRAS to diagnose individual “at-risk” for myopia progression was compared against decision of five clinicians in 149 myopes, aged 6–29 years. Using model-1 (no-weightage), further 7 sub-models were created with varying number of risk factors in decreasing step-wise manner (1a: 10 factors to 1g: 4 factors). In random eye analysis for model-1, the highest Youden’s J-index (0.63–0.65) led to the MPRAS cut-off score of 41.50–43.50 for 5 clinicians with a sensitivity ranging from 78 to 85% and specificity ranging from 79 to 87%. For this cut-off score, the mean area under the curve (AUC) between clinicians and the MPRAS model ranged from 0.89 to 0.90. Model-2 (weighted for few risk-factors) provided similar sensitivity, specificity, and AUC. Sub-model analysis revealed greater AUC with high sensitivity (89%) and specificity (94%) in model-1g that has 4 risk factors compared to other sub-models (1a–1f). All the MPRAS models showed good agreement with the clinician’s decision in identifying individuals “at-risk” for myopia progression.

## Introduction

The global prevalence of myopia is estimated to rise to 5 billion by the year 2050, among which, nearly 1 billion individuals are likely to progresses to high myopia^1–5^. Myopia progression rate in children is reported to vary between − 0.09 to − 1.20 D/year among different ethnicities, with a considerable number of children undergoing rapid myopia progression (> 1 dioptre/year)^6–9^. Slowing down the myopia progression by at least one dioptre during childhood should lower the risk of developing myopic maculopathy by 40% in both low and high myopes throughout their lifetime^10^. Determining the risk level of myopia progression at the earliest can play a critical role in myopia management as it helps in recommending appropriate myopia control strategies, decide the follow-up to monitor changes in axial length and/or refraction, and help in preventing associated visually debilitating complications.

Inspite of an increase in the awareness about myopia and the importance of its management with various myopia control strategies, the practice pattern for managing myopia remained unaltered among eye care practitioners in the continents such as Australasia, Asia, Europe, North and South America^11^. It was reported that the number of practitioners concerned about the increasing frequency of pediatric myopia in their practices was highest in Asia and lowest in Australasia. Despite an increase in the prevalence of myopia as well as increased concern about the perceived clinical practice, refractive correction of myopia still includes the prescription of either single-vision spectacles/contact lenses and under correction of single vision distance correction by the practitioners worldwide, even if the individual is at risk of undergoing myopia progression^11–13^. Recent evidence from Spain and Africa also indicated a similar trend and reported that reasons for such practice patterns are related to the cost of myopia control treatment options, inadequate information, lack of training, and unpredictable outcomes about the myopia control options^13,14^. A global survey revealed that about 50% of pediatric ophthalmologists had a dilemma to initiate or prescribe appropriate myopia control treatment, beyond prescribing single vision lenses^15^. This indicates the need for developing a simple risk stratification system, primarily for two main purposes: (1) to identify children “at-risk” for myopia progression, and (2) to make children and their parents understand the necessity of myopia management. The former is advantageous to the clinician in decision-making about starting or changing the myopia control treatment options, and the latter is beneficial to the patient/parent for compliance in their treatment approach.

Considering that myopia development and its progression underpins a complex multifactorial etiology that interplays between parental myopia^16–19^, environmental (near work activities and time spent in outdoors)^20–28^, and other known potential risk factors^7,29–32^ (such as earlier age of myopia onset^7,28–30,33^, relative peripheral hyperopia^34–37^, near esophoria^38,39^, and high lag of accommodation^39–41^), the better way to determine the level of risk for myopia progression in an individual is by assessing these risk factors through a holistic approach.

Three related works are worth highlighting: (1) The Brien Holden Vision Institute (BHVI)’s myopia calculator (https://bhvi.org/myopia-calculator-resources/); (2) Du et al.’s risk score model^42^; and (3) The Ulster University’s model for Predicting Myopia Onset and Progression (PreMO)^43^. While the myopia calculator only indicates how different myopia control strategies would work on a specific individual when age, gender, and ethnicity data are provided, Du et al. score model enables the identification of myopic symptoms and is not aimed at the determination of risk for myopia progression. The PreMO model helps to identify the likely age of myopia onset based on 3 myopic risk parameters and the risk of myopia progression based on refractive error change and age alone. Although timely intervention slows myopia progression and reduces the risk of developing ocular pathologic complications, there is no risk scoring system available that captures detailed risk factors to identify the individuals “at-risk” for myopia progression by considering multiple risk factors. Having such a scoring system based on multiple risk factors is likely to not just help in identification of “at-risk” individuals, but also to decide on appropriate myopia control strategies. Therefore, the aim of this study was to develop a risk score system—“myopia progression risk assessment score” (MPRAS) combining multiple known risk factors including parental myopia, environmental, and optical factors to identify individuals “at-risk” for myopia progression.

## Methodology

The study was approved by the Institutional Review Board of L V Prasad Eye Institute (LVPEI), India (Ethics Ref: LEC 04-17-029), and adhered to the tenets of the Declaration of Helsinki. All patients had signed the general written informed consent before their clinical evaluation approving the use of their data for research purposes. The parents/guardian signed the informed consent if the individual was less than 18 years of age.

**Figure 1:**
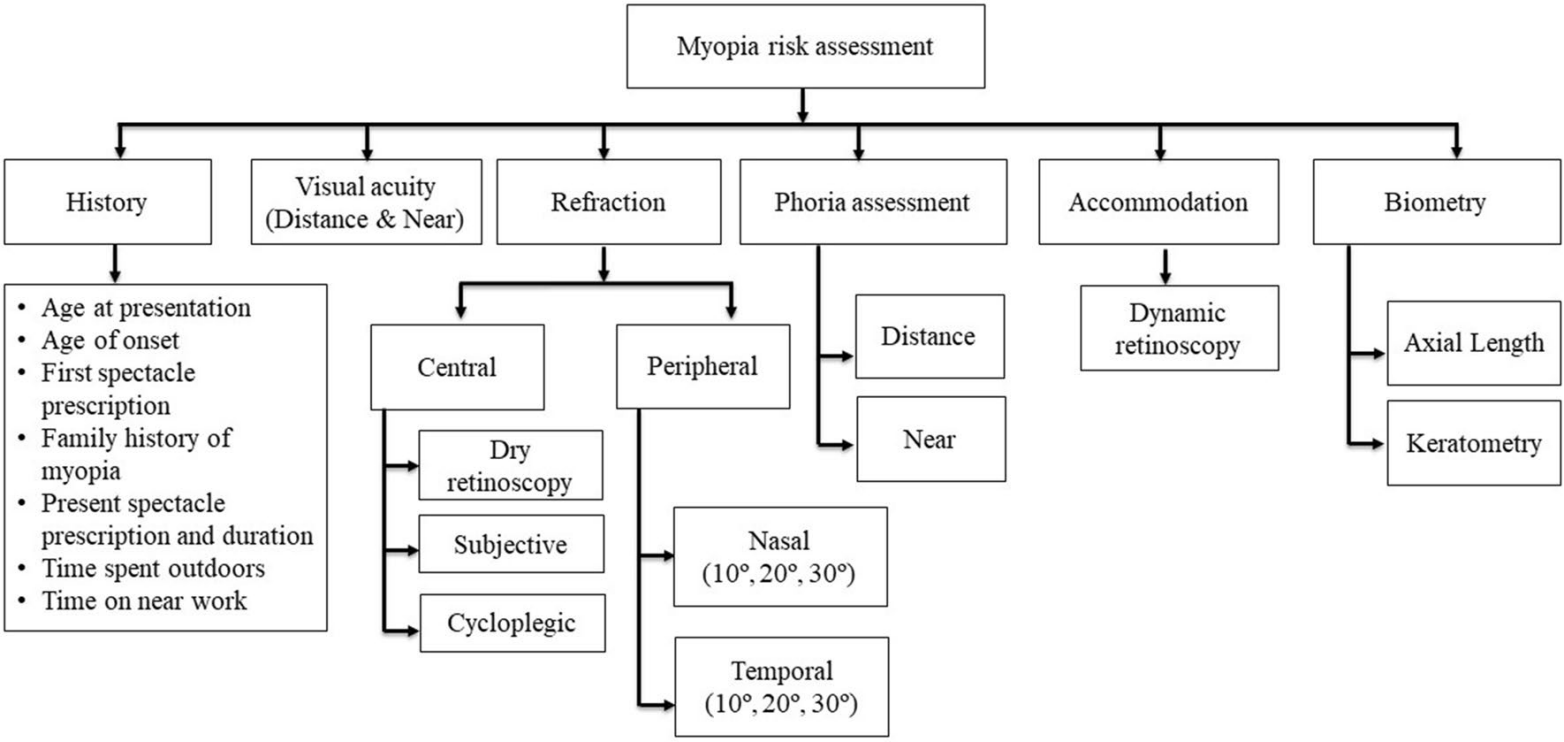
Flow chart demonstrating the procedures conducted at the Myopia Centre of LVPEI.

The MPRAS model was built based on myopia-related information retrieved from the electronic medical records (EMR) of 149 consecutive patients (with myopic refractive error) who visited the “Infor Myopia Centre” of LVPEI, India during year 2020–2021. All the individuals with myopia (spherical equivalent refraction (SER) ≤ − 0.50D), underwent a specialty eye examination to determine various risk factors associated with myopia progression (Fig. 1). A structured examination protocol was used to gather a detailed history of a patient that included the age at presentation (current age of the individual during the visit to the clinic), myopia onset age (age at which the individuals started to wear spectacle/contact lenses for the first time to correct myopic refractive error), and the number of myopic parents (either father or mother, or both parents), and information related to environmental factors such as the number of hours per day spent in outdoors and all the near work activities (i.e., a handheld electronic device such as smartphone, tablets, reading and writing on printed material for either academic and non-academic purposes, drawing, painting, and crafting). In the optical factors, change in spectacle prescription (the refraction at current eye examination visit subtracted from the refraction value obtained 1-year ago during previous examination) and/or axial length (the central axial length at current eye examination subtracted from the axial length value obtained 1-year ago during previous examination), current central cycloplegic refraction, relative peripheral refraction (relative peripheral refraction was determined by subtracting the values of central refraction from peripheral refraction), accommodative error (lag/lead), and phoria status at distance and near were recorded. Cycloplegic refraction was performed after 40 min of the one drop of 1% cyclopentolate ophthalmic solution (CYCLOFEZ, ENTOD, India) instillation in both eyes to decide the final spectacle prescription.

Axial length was determined with a non-contact biometer (LS 900, Haag Streit, Switzerland or IOL Master 700, Carl Zeiss Meditec Ltd, Germany). Peripheral refraction was determined along the horizontal meridian (180 degrees) until ± 30° in 10° intervals using an open-field autorefractor (Shin-Nippon N-Vision K 5001, Japan) based on the method described by Verkicharla et al.^44^. For the purpose of risk assessment, we used the peripheral refraction values at ± 30° eccentricity. Relative peripheral hyperopia of at least 0.25 D in magnitude along either the nasal or temporal meridian was considered for risk score models. The phoria status was assessed using the modified Thorington test and the accommodative error was determined using monocular estimation method of dynamic retinoscopy (performed at 40 cm). Based on all the parameters related to parental myopia, environmental, and optical factors, clinician diagnosed the individual to one of the categories, i.e., either “at-risk” for myopia progression (expecting the change in myopic refractive error of ≥ 0.50 D prospectively in 1 year) or “low-risk” for myopia progression (expecting the change in myopic refractive error of < 0.50 D prospectively in 1 year). The clinical decision served as a gold standard for this study. Individuals who had non-axial myopia such as myopia associated with keratoconus, lenticonus, and micro-spherophakia, pathologic myopia lesions, and any systemic/ocular conditions, that could influence the state of refractive error were excluded from the study.

### Development of myopia progression risk assessment score (MPRAS)

After a thorough assessment of the literature^7,16–26,29–41,45–71^ and multiple focused group discussions among six clinicians who have seen at least 500 patients in the Myopia Centre, a list comprising ten known risk factors (parental myopia, environmental, and optical factors) associated with the development and progression of myopia was prepared (Table 1, same list is used for the clinical decision making). According to sample size calculation, data of 132 patients (risk cases: 101, low-risk cases: 31) were necessary to obtain an area under the Receiver Operating Curve (ROC) of 0.90. A null hypothesis value of AUC 0.80 was used with a 5% level of significance and 80% power.

**Table 1:** Myopia risk assessment protocol (model-1) incorporating various risk factors categorized into low, intermediate, and high-risk.

Sl. No.	Myopia risk factors	Low-risk (Score: 1)	Intermediate-risk (Score: 2)	High-risk (Score: 3)
1	Change in SER or AL (D or mm change/year)^7,29–32,45–48^	> − 0.50 or < 0.10	− 0.50 to − 0.75 or ≥ 0.10 to < 0.20	< − 0.75 or ≥ 0.20
2	Age at presentation (years)^7,29,30,33^	≥ 18	> 13 to < 18	≤ 13
3	SER (D) at presentation^7,29–32,45–47^	≥ − 3.00	< − 3.00 to > − 6.00	≤ − 6.00
4	Age of onset (years)^7,29,30,33^	≥ 18	> 13 to < 18	≤ 13
5	Time spent outdoors (hours per day)^20–23,49–55^	> 2	1–2	< 1
6	RPR at presentation (Hyperopic defocus (D))^34–37,56^	< + 0.25	≥ + 0.25 to + 0.50	> + 0.50
7	Time spent on near work (hours per day)^24–26,57–66^	≤ 7	> 7 to 10	> 10
8	Near esophoria (PD)^38,39^	≤ 2	> 2 to < 6	≥ 6
9	Accommodative lag (D)^39–41,67^	≤ + 0.50	+ 0.75 to + 1.00	> + 1.00
10	Myopic parents^16–19,51,68–71^	0	1	2

*SER* spherical equivalent refractive error, *AL* axial length, *RPR* relative peripheral refraction, *D* dioptre, *mm* millimeter, *PD* prism diopter, Change in SER—the refraction at current eye examination subtracted from the refraction (or spectacle prescription) value obtained 1-year ago during previous examination, Change in AL—the central axial length at current eye examination subtracted from the axial length value obtained 1-year ago during previous examination.

In total, 149 individuals with myopia presented to our Myopia Centre in 2020–2021 (consecutive patients), in which 35 individuals were at “low-risk” for myopia progression and 114 were categorized as “at-risk” cases. Of the total 149 myopes, 48% of myopes were male and 52% were female. The patients who visited the myopia clinic for the specialty evaluation were from different states of India. All the cases were graded by 5 masked clinicians at the Myopia Centre to receive their clinical decision on whether they were at “low-risk” or “at-risk” of myopia progression. All the clinicians were masked and unaware of each other’s clinical decisions. From the clinical point of view, we advise the patients to follow-up with the myopia clinic after 6 months to monitor biometry and refraction to decide on further treatment management for the myopia progression.

Two risk models were created: one model with an increasing level of weightage given to one or more individual risk factors based on the permutation importance of the myopic risk factors from the XGBoost algorithm (Fig. 4) and another model with no weightage (model-1: no-weightage, and model-2: weightage). The risk scoring system for model-1 is shown in Table 1, and information related to model-2 is given separately as supplementary Table S1. Considering the literature review and multiple deliberations amongst the group involved in developing the risk score, 10 risk factors (continuous variables) were identified. For each individual, based on the severity of risk assessed through the numerical information, each of the assessed 10 factors are assigned to either low, intermediate, or high-risk category as shown in Table 1. Risk assessments were performed for the right eye, left eye, and random eye (selected either right eye or left eye based on the randomization performed in Microsoft Excel 2016) of 149 myopic individuals, separately.

Following one of the most accepted risk score models in ophthalmology—“Convergence Insufficiency Symptom Survey” (CISS) score^72^, points of 1, 2, and 3 were assigned to each of the risk factors in the MPRAS. For model-1, low, intermediate, and high-risk factors were assigned a value score of 1-point, 2-points, and 3-points, respectively. First, all the low-risk values, intermediate-risk values, and high-risk values were summed up individually for all the risk parameters of myopia progression. Second, the total score of low-risk value was multiplied by 1, intermediate risk value by 2, and high-risk value by 3. Finally, the total “myopia progression risk assessment score” was obtained by adding the individual point scores from the entire level of risk categories (low-risk, intermediate-risk, and high-risk). Based on the final score which could range from 10 to 90 points, patients were categorized either in the “low-risk” or “at-risk” category.

Based on the previous literature^7,29,73^ and also considering the risk factors that have a greater impact on identifying the risk of myopia progression compared to other risk parameters among the 10 listed factors in the current study, we have given an extra weightage to a few risk factors to understand how each model (model-1 and -2) vary with the change in cut-off score. In model-2, an extra weightage (2×) was given to the change in refractive error per year for the intermediate (2 × 2 points = 4) and high-risk values (2 × 3 points = 6). In addition, if age is ≥ 18 years, the points for (a) the number of myopic parents and (b) time spent outdoors were reduced by 0.5 times in the intermediate and high-risk categories (i.e., 0.5 × 2 points = 1 for intermediate, and 0.5 × 3 points = 1.5 for high-risk values) given that these risk factors were less likely to affect the progression of myopia during adulthood^74,75^. Examples for model-1 are shown in the supplementary Table S2.

### Agreement between clinician decision and machine learning algorithm

In addition, we used XGBoost (Extreme Gradient Boosting) algorithm to identify the permutation feature importance of the myopia risk factors that we used in this MPRAS model to investigate how much the model depends on specific feature. XGBoost is a decision tree-based ensemble machine learning algorithm which helps to accurately predict a target variable (unstructured data) by ensemble learning method that combines a set of weak learners into a strong learner^76,77^. In order to implement this XGBoost model, first we used the XGBClassifier method to train the XGBoost model^78^, which is followed by permutation importance^79^.

Based on permutation feature importance of risk factors used in model-1, we have further created sub-models (1a–1g) by decreasing the number of risk factors in a step-wise manner (Table 2) with 1a containing all the 10 risk factors, and 1g containing only 4 risk factors. The rationale behind creating sub-models is to investigate how the clinical judgment agrees/varies with the MPRAS model with limited risk factors or in case any of the parameters are not available during the clinical eye examination.

**Table 2:** List of minimum parameters that can be used in the clinic to identify the myopic individual who is “at-risk” of myopia progression.

(1a) 10 Risk parameters	(1b) 9 Risk parameters	(1c) 8 Risk parameters	(1d) 7 Risk parameters	(1e) 6 Risk parameters	(1f) 5 Risk parameters	(1g) 4 Risk parameters
Change in SER or AL	Change in SER or AL	Change in SER or AL	Change in SER or AL	Change in SER or AL	Change in SER or AL	Change in SER or AL
Age at presentation	Age at presentation	Age at presentation	Age at presentation	Age at presentation	Age at presentation	Age at presentation
SER at presentation	SER at presentation	SER at presentation	SER at presentation	SER at presentation	SER at presentation	SER at presentation
Age of onset	Age of onset	Age of onset	Age of onset	Age of onset	Age of onset	Age of onset
Time spent outdoors	Time spent outdoors	Time spent outdoors	Time spent outdoors	Time spent outdoors	Time spent outdoors	
RPR at presentation	RPR at presentation	RPR at presentation	RPR at presentation	RPR at presentation		
Time spent on near work	Time spent on near work	Time spent on near work	Time spent on near work			
Near esophoria	Near esophoria	Near esophoria				
Accommodative lag	Accommodative lag					
Myopic parents						

*SER* spherical equivalent refractive error, *AL* axial length, *RPR* relative peripheral refraction.

Taking the feedback from the XGBoost algorithm, area under the curves and ROC analysis were performed separately for all the sub-models 1a–1g. Principal components analysis was also performed on 10 listed risk factors to determine the minimum independent risk factors required to identify the risk of myopia progression.

### Agreement between clinician decision and diagnostic test using 1-year follow-up data (retrospective data)

A separate analysis was conducted to investigate the agreement of clinician's decision with the objective diagnostic test i.e., change in refractive error determined using retinoscopy. For this, another set of data of 132 myopes who visited at least twice to any of the out-patient-departments of LVPEI over a period of 1 year was considered. This data used for validation is independent of the data used for developing the model as the later needed extra information that was available through the patients seen only in Myopia Centre. The follow-up duration of each participant was determined based on the number of days between each visit (i.e., 335–390 days gap between each visit was considered for a 1-year progression). Based on a total of four risk factors (change in refractive error, age at presentation, refractive error at presentation, and age of onset) that were recorded as a part of routine eye examination in baseline visit, clinician-1 and clinician-6 (PKV and MKM) retrospectively classified individuals under “at-risk” or “low-risk” category of myopia progression. Change in myopic SER of ≥ 0.50 D/year (non-cycloplegic value) was considered “at-risk” of myopia progression as the diagnostic criteria (the gold standard in this case) and was compared against the clinician's decision.

### Validation of MPRAS model decision with change in refractive error after 1-year prospectively

To validate the MPRAS model decision with a longitudinal change in refractive error (1-year) prospectively, we used a subset of 36 individuals with myopia (of 149 myopes) who did not use myopia control interventions for 1 year after the decision made with MPRAS model. The individuals who had the change in myopic refraction (cycloplegic value) of ≥ 0.50 D/year (between 335 and 390 days gap) prospectively were labelled into myopia progression and were compared against the decision of the MPRAS model’s assessment that was made with all 10 risk factors at the baseline visit.

### Statistical analysis

The statistical analyses were performed using Microsoft Office Excel 2016 (Microsoft Cooperation, USA) and IBM SPSS® Statistics 21.0 (SPSS, USA). All the 10 risk parameters were used for the MPRAS model to obtain the total risk score and then compared with each clinician’s decision. The ROC analysis was used to estimate the coordinates of the curve such as sensitivity, specificity, area under the curve (AUC), and point score values of the MPRAS model. The area under the ROC curve was used to determine the overall ability of the MPRAS score to differentiate between those with “low-risk” or “at-risk” of myopia progression. The cut-off point for the score was determined using Youden’s J-index^80^. Cohen's Kappa^81^ was used to compare the inter-rater reliability within the clinical decisions of five clinicians. Principal components analysis was performed to identify the independent components from the 10 myopia risk factors included in this study.

## Results

**Figure 2:**
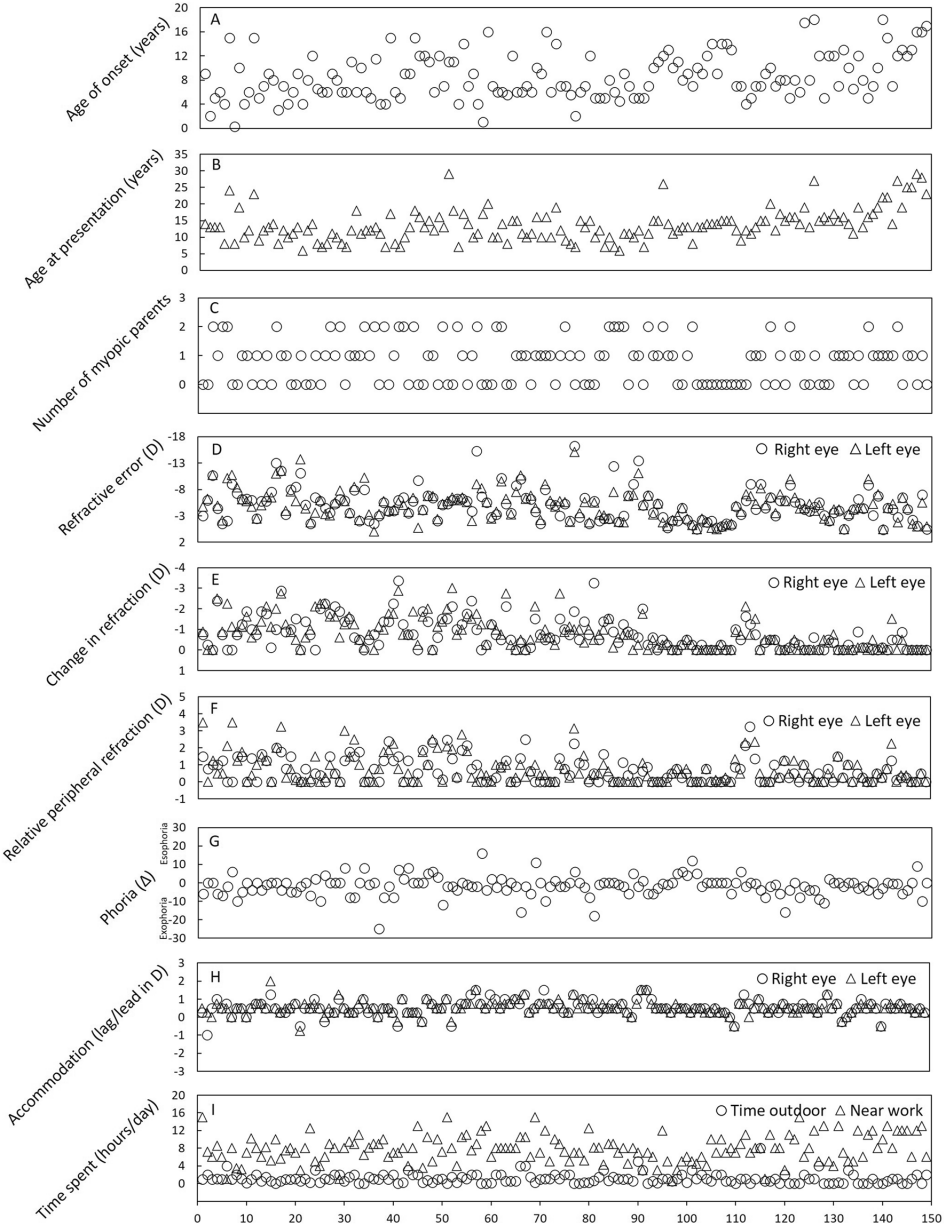
Distribution of (**A**) age of onset, (**B**) age at presentation, (**C**) the number of myopic parents, (**D**) spherical equivalent refractive error, (**E**) change in refractive error in 1 year, (**F**) relative peripheral refraction, (**G**) status of near phoria (+ and − sign indicate esophoria and exophoria, respectively), (**H**) accommodative error (+ and − indicate lag and lead of accommodation, respectively), and (**I**) time spent on near work and outdoor.

Figure 2 shows the distribution of 149 myopic individuals based on risk parameters of myopia progression. The mean ± SD (range) age of myopia onset and age at presentation of 149 myopic individuals were 8.59 ± 3.74 (0.3–18) and 13.74 ± 4.89 (6–29) years, respectively. Likewise, the mean SER was − 5.02 ± 2.99 D and − 5.00 ± 2.82 D for the right and left eyes, respectively. The mean annual myopia progression in the right eye and left eye was − 0.73 ± 0.78 D and − 0.74 ± 0.84 D, respectively. The average number of hours spent on near-work activities was 7.85 ± 3.05 h/day and in outdoor activities was 1.11 ± 1.14 h/day. The number of children with 0, 1, and 2 myopic parents were 61, 59, and 29, respectively. The mean accommodative error was + 0.53 ± 0.42 D and + 0.52 ± 0.38 D for the right and left eyes, respectively. The mean relative peripheral refraction was + 0.66 ± 0.71 D and + 0.75 ± 0.91 D for the right eye and left eye, respectively. Near phoria ranged from esophoria 16 Δ to exophoria − 25 Δ.

The clinical decision among the five clinicians at the Myopia Centre showed strong agreement with the Cohen's Kappa ranging from 0.90 to 1.00. For the right eye analysis, clinical judgment of 4 clinicians in comparison with clinician 1 showed sensitivity of 96–100% and specificity of 97–100%, and for the left eye analysis, sensitivity of 99–100% and specificity of 91–97% was found, suggesting a minimal inter-clinician variability in clinical judgment.

### Sensitivity and specificity of 2 MPRAS models

**Figure 3:**
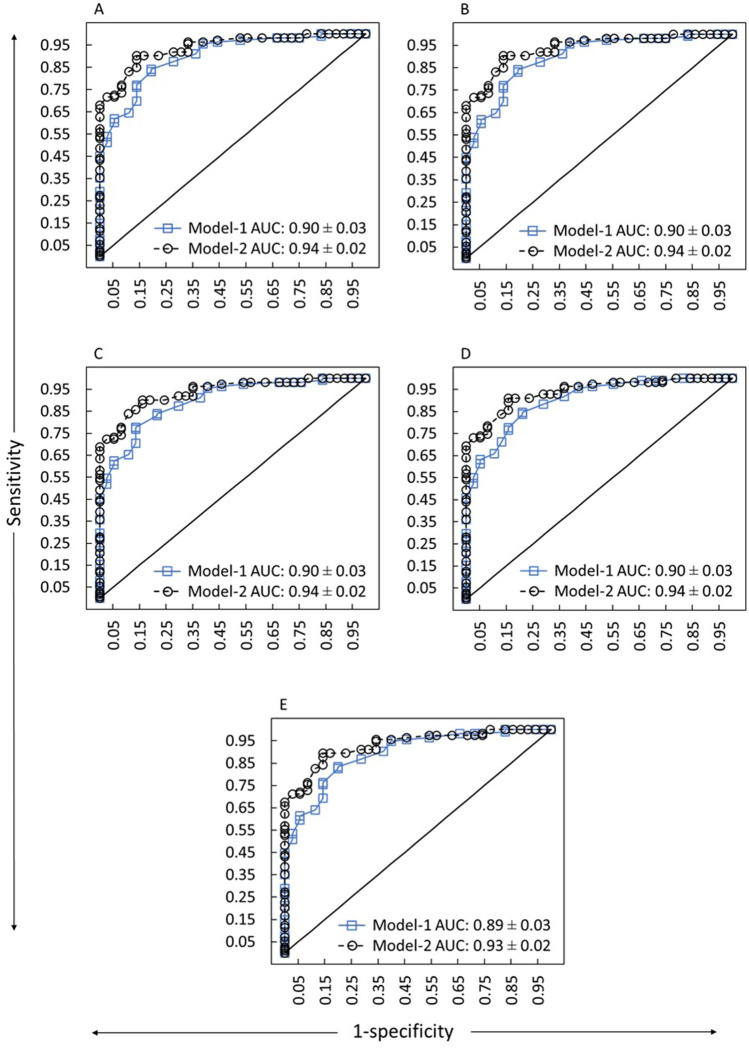
Receiver operating characteristics (ROC) curves for two different models compared against the clinical decisions of five clinicians at the Myopia Centre. Panel (**A**–**E**) indicate for the clinician C1, C2, C3, C4, and C5, respectively. Data is based on random eye analysis.

Based on the random eye analysis, the ROC curves of two distinct risk score models compared against the clinical decisions of 5 clinicians are shown in Fig. 3. The mean AUC for the two risk score models ranged from 0.89 to 0.94. For model-1, in random eye analysis, the highest Youden’s J-index (0.63–0.65) led to the MPRAS cut-off score of 41.50 for 4 clinicians and 43.50 for 1 clinician with sensitivity ranging from 78 to 85% and specificity ranging from 79 to 87%. Individual clinician’s cut-off score, sensitivity, specificity, AUC, and Youden’s J-index for the right, left, and random eye are shown in Table 3. The MPRAS for 12% (18/149) of the individuals was below the cut-off value (43.50) for the myopes “at-risk” for progression per the clinical judgment.

**Table 3:** The Youden’s J-index, sensitivity, specificity, AUC, and cut-off values of the MPRAS model-1 for the clinical decision of five clinicians at the Myopia Centre.

	Youden's J-index	Sensitivity	Specificity	AUC	Cut-off
Right eye					
C1	0.61	0.72	0.89	0.88 ± 0.03	43.50
C2	0.61	0.72	0.89	0.88 ± 0.03	43.50
C3	0.61	0.73	0.89	0.88 ± 0.03	43.50
C4	0.60	0.73	0.87	0.88 ± 0.03	43.50
C5	0.57	0.71	0.86	0.87 ± 0.03	43.50
Left eye					
C1	0.68	0.79	0.89	0.91 ± 0.03	43.50
C2	0.67	0.78	0.89	0.91 ± 0.03	43.50
C3	0.66	0.77	0.89	0.90 ± 0.03	43.50
C4	0.66	0.77	0.89	0.90 ± 0.03	43.50
C5	0.65	0.77	0.88	0.89 ± 0.03	43.50
Random eye					
C1	0.65	0.84	0.81	0.90 ± 0.03	41.50
C2	0.65	0.84	0.81	0.90 ± 0.03	41.50
C3	0.64	0.78	0.87	0.90 ± 0.03	43.50
C4	0.64	0.85	0.79	0.90 ± 0.03	41.50
C5	0.63	0.83	0.80	0.89 ± 0.03	41.50

In this table, C1, C2, C3, C4, and C5 represent the first, second, third, fourth, and fifth clinicians, respectively.

For model-2, a cut-off score of 41.75 yielded the highest Youden’s J-index (0.74–0.76) for all clinicians with maximum sensitivity (ranging from 89 to 91%) and specificity (84–86%). Model-1 and -2 led to a similar cut-off value irrespective of extra weightage given for the risk factors. More details about Youden’s J-index, sensitivity, and specificity of the models in comparison with all clinicians are given in Supplementary Table S3.

### Agreement between clinician decision and machine learning algorithm

**Figure 4:**
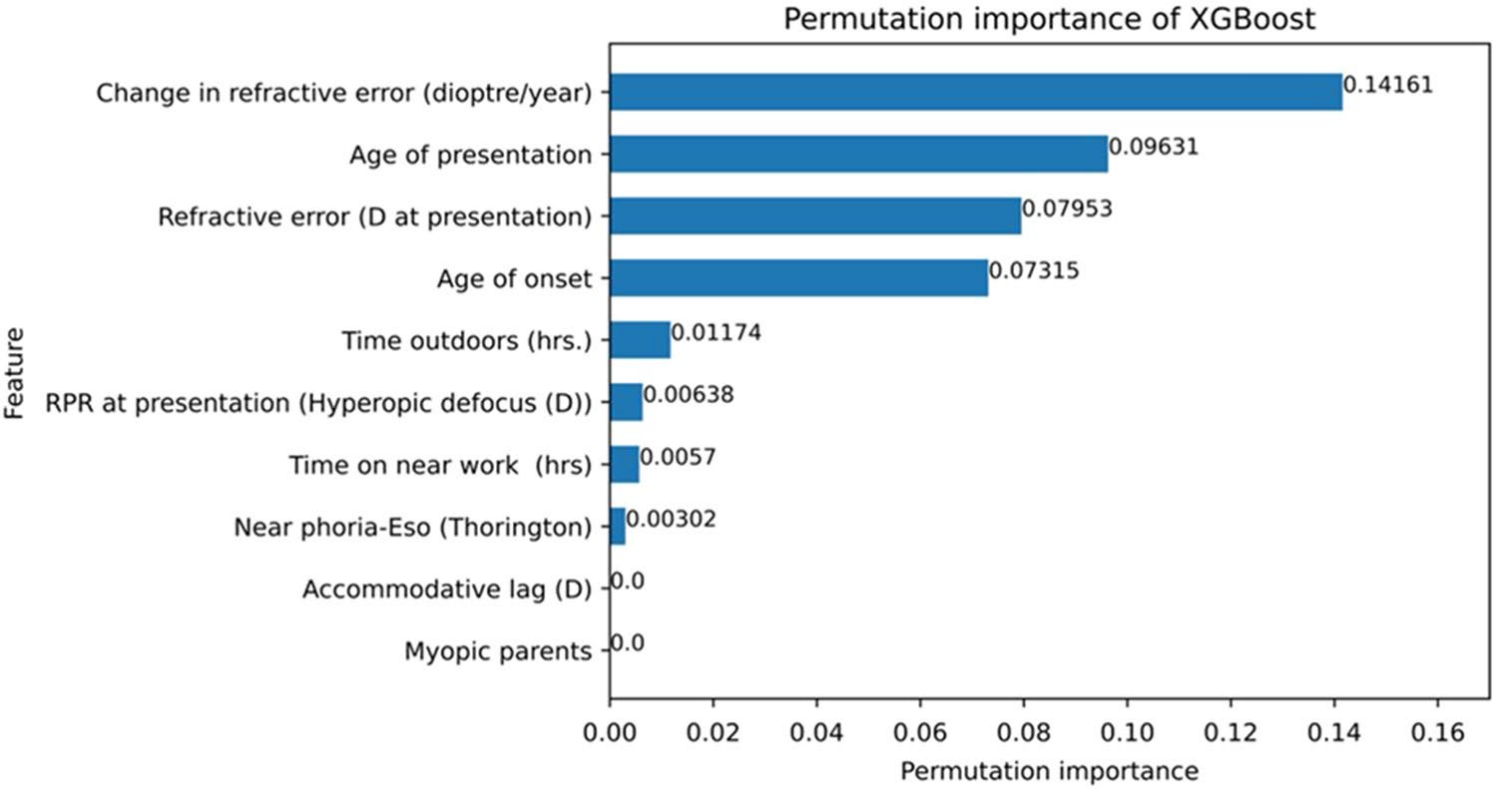
Feature importance of myopic progression risk factors data using permutation importance of a feature.

Figure 4 shows the bar graph of feature importance for the listed risk factors of myopia progression. Of the 10 myopic risk factors, the most important factors were the change in refractive error (0.14), age at presentation (0.09), refractive error at presentation (0.07), and age of onset (0.07) compared to other myopic risk factors.

Using model-1, six additional ROC analyses were performed with the gradual removal of risk parameters (i.e., number of myopic parents, accommodative error, near esophoria, near work, relative peripheral refraction, and time outdoor). A cut-off score ranging from 41.50 to 22.00 yielded the highest Youden’s J-index (0.65–0.84) for “clinician 1” with sensitivity range of 74–89% and with specificity range of 81–94%. The AUC remained identical irrespective of the number of risk factors included in the model-1a to -1f (i.e., model-1a with 10 risk factors to model-1f with 5 risk factors). However, model-1g showed a relatively greater AUC value compared to other sub-models (1a–1f). The eigenvalues of the principal components analysis were greater than 1 for only 4 myopic risk factors (i.e., change in refractive error, age at presentation, refractive error at presentation, and age of onset) among 10 listed factors. The Youden’s J-index, sensitivity, specificity, AUC, and cut-off score with 10 to 4 risk parameters are shown in Table 4.

**Table 4:** The Youden’s J-index, sensitivity, specificity, AUC, and cut-off values of the MPRAS model-1 with stepwise removal of myopic risk parameters.

	Model-1	Youden's J-index	Sensitivity	Specificity	AUC	Cut-off
1a	10 parameters	0.65	0.84	0.81	0.90 ± 0.03	41.50
1b	9 parameters	0.68	0.82	0.86	0.90 ± 0.03	39.50
1c	8 parameters	0.68	0.76	0.92	0.90 ± 0.03	38.50
1d	7 parameters	0.65	0.74	0.92	0.89 ± 0.03	37.50
1e	6 parameters	0.68	0.76	0.92	0.92 ± 0.02	33.50
1f	5 parameters	0.68	0.79	0.89	0.92 ± 0.02	29.50
1g	4 parameters	0.84	0.89	0.94	0.96 ± 0.01	22.00

### Agreement between clinician decision and diagnostic test using 1-year follow-up data

Based on a separate retrospective mode of analysis of data from 132 individuals, clinician's (PKV and MKM) decision showed sensitivity of 88–95% and specificity of 59–74% in picking up the individual's “at-risk” for myopia progression. Only 2–5% of myopic individuals who were evaluated as having “low-risk” of myopia progression at the baseline visit showed an increase in myopic refractive error of ≥ 0.50 D in 1 year.

### Validation of MPRAS model decision with change in refractive error after 1-year prospectively

Based on the prospective change in refractive error in a subset of 36 individuals with myopia, we found that the MPRAS model decision was correct (i.e., either the individuals with myopia is “at-risk” or “low-risk” of myopia progression) for the 75% (n = 27/36) of individuals with myopia at the baseline visit. In addition, in this subset, the MPRAS model decision showed sensitivity of 94% and specificity of 60% in identifying the individuals with myopia “at-risk” for myopia progression based on the change in refractive error prospectively.

## Discussion

We report two novel risk scoring models that are sensitive to identifying an individual with a myopic refractive error “at-risk” for myopia progression and have immense potential in myopia management.

Assessing various risk factors, risk stratification models are the critical components of myopia management practice and MPRAS can form an integral part of the current practice pattern. Du and colleagues developed a risk score model to predict myopia symptoms in 6–19 years old students after incorporating several environmental risk factors^42^. However, due to the heterogeneous etiology of myopia, inclusion of factors other than the environmental factors can better predict/identify the risk of myopia progression in patients^28,37,82–84^. The BHVI myopia calculator is another tool that combines patient data (age, ethnicity, current prescription) with various optical and pharmaceutical treatment options to show the impact on the myopia progression rate with and without treatment.

The higher score value of MPRAS (> 41.50 for model-1) can be considered as an indicator to treat patients aggressively and clinicians could select an efficient treatment strategy and schedule appropriate follow-up visits to monitor the progression. A similar optimal cut-off score was found with both the non-weighted model-1 (for 4 clinicians > 41.50 and for 1 clinician > 43.50) and weighted model-2 (> 41.75). Considering the better sensitivity and specificity and ease in using, we advocate using model-1 with a cut-off score of > 41.50 to administer in clinical settings. The majority of risk factors included in MPRAS models can be collected as a part of routine clinical evaluation, nevertheless, the question which we do not want to overlook was: How does the MPRAS model will function when a few of the risk factors are missing? Our investigation showed that a model-1 with as low as five risk factors to a much more sophisticated one with ten risk factors can help in identification of individuals “at-risk” for myopia progression where AUC remains similar (Table 4). In fact, model-1g involving 4 parameters (change in refractive error, age at presentation, refractive error at presentation, and age of onset) showed relatively greater AUC with maximum sensitivity and specificity to identify the risk of myopia progression compared to other sub-models (1a–1f). The eigenvalues from the principal components analysis also indicate that a minimum of 4 risk factors is only required to identify the risk of myopia progression. While a four-factor model can be utilized to identify children at risk of myopia progression, documenting multiple risk factors could aid in better management of myopia. Unlike previously proposed models^42,43^, which cannot help to decide the appropriate myopia control treatment strategies, the current MPRAS model helps to understand multiple risk parameters (10 risk factors) and also likely to aid in deciding the appropriate/suitable myopia control treatment strategies to counteract the myopia progression.

Instruments to measure ocular biometry and/or peripheral refraction are still less accessible to many clinical practices^11,12^. We recommend using the refraction values in the form of spherical equivalent in cases when axial length measurement is not available. Secondly, there is evidence indicating that peripheral hyperopic defocus alters ocular growth^85–87^. Even though the link between peripheral refraction and myopia development/progression is controversial, several recent corrective modalities/myopia control lenses work on counteracting the relative peripheral hyperopic defocus to control myopia progression^88–91^. Recent evidence from Zhang and colleagues reported that children who used the Defocus Incorporated Multiple Segments (DIMS) spectacles with baseline relative peripheral hyperopia showed less myopia progression and less axial elongation than children with baseline relative peripheral myopia, which suggests the role of counteracting hyperopic defocus in the peripheral retina to control the progression of myopia^92^. Thus, we believe that determining peripheral refraction in at least one possible retinal eccentricity in the horizontal meridian (> 20°) might help the clinician in understanding the magnitude and profile of relative peripheral defocus. In turn, relative peripheral refraction would also aid in the selection of a treatment plan in the form of peripheral defocus spectacles or contact lenses. If the peripheral defocus is already more myopic than the central values (relative peripheral myopia), there may not be a need for prescribing peripheral defocus lenses, instead, other strategies can be recommended and vice-versa^92^, this needs to be investigated further in detail in future. Regarding environmental factors, details related to outdoor activities such as timings of outdoor activities, the number of hours, use of hat/cap/sunglasses during outdoor activities, location, and weekdays/weekend differences might be useful in providing appropriate recommendations in the form of “environmental strategies”. While evidence started to appear indicating the clinically insignificant effect of time outdoors on controlling myopia progression (in existing myopes), we still included time outdoors as a factor in the MPRAS model for following 2 reasons and to avoid eliminating one of the modifiable risk factors: (1) this model can also be applied to emmetropic or pre-myopic groups, where time outdoors is considered protective for myopia and (2) benefit of the doubt given to the time outdoors for its negative effect as reported by Cao et al. in a systematic review and meta-analysis that more time spent outdoors helped to control the axial elongation, thereby reducing the risk of myopia progression in children^93^.

The strength of the MPRAS model is that it included individuals with different magnitudes of myopia (− 0.50 to − 16.50 D) and the age range from 6 to 29 years. There are certain limitations for the current study. Patients were seen in the Myopia Centre between 1st November 2020 and 30th April 2021 and this time period typically occurred after the lockdown period of the COVID-19 pandemic in India. However, while the influence of the pandemic and seasons (maybe) on the myopia progression in myopes cannot be ruled out, it can be expected that the other risk factors also showed some associated changes. For example—more near work and less time outdoors, greater changes in axial length, etc. are associated with more myopia progression. On the positive side, considering that the entire data was captured within a span of 6 months (non-rainy seasons in India), the data should potentially eliminate the confounding effect of seasons on myopia progression and thus leading to controlled data set. The risk factors for myopia progression of the model were obtained from the consensus among clinicians based on evidence from published literature. In addition, we also used XGBoost algorithm to understand the feature importance of risk factors of myopia progression in this study. The MPRAS model was assessed for its agreement with only the clinician's decision (in a cross-sectional design manner), but further investigation is underway to validate the MPRAS model based on longitudinal observation to see whether the myopic individuals exhibit myopia progression or not as predicted by the MPRAS model. Through this research, we establish the proof-of-concept that can be further extended to include more data and develop an application. While our retrospective analysis validating the clinician's decision with the diagnostic test indicates good sensitivity, we used data from only 4 risk factors (change in refractive error, age at presentation, refractive error at presentation, and age of onset) that were captured from the routine clinical examination that also includes refraction with non-cycloplegic autorefraction. In addition, we validated the MPRAS model decision against the prospective change in refractive error in a subset of 36 individuals with myopia. Considering that individuals with high degrees of myopia can experience myopia progression even in adulthood, we included high myopes in the analysis. We had only 7 myopes with refractive error worse than − 10 D and recommend future studies to include larger sample of severe myopes (< − 10.00 D) in risk score models. The current model aims to identify the children “at-risk” for myopia progression, however, future studies with large sample are needed to investigate model for identifying the risk of myopia progression based on rate of progression as slow or fast progressors.

## Conclusion

In this study, we developed a novel myopia risk score assessment model for the clinicians to identify if patients with myopia are “at-risk” or “low-risk” for myopia progression. While the two proposed MPRAS models showed a good agreement with the clinicians in identifying individuals at risk for myopia progression, we propose the use of model-1 based on the sensitivity and specificity along with its simplistic nature in the application. This MPRAS system along with the professional clinical decision will assist clinicians in their myopia management strategies, as well as improve parental understanding related to their child’s risk of myopia progression.

## Supplementary Information

Supplementary Tables.

## Data Availability

The data that support the findings in this study are available from the corresponding author on reasonable request.
