# Supplementary material for: Myopia progression risk assessment score (MPRAS): a promising new tool for risk stratification
Source: Sci Rep. 2023 May 31;13:8858. doi: 10.1038/s41598-023-35696-2 (PMC10231302; doi:10.1038/s41598-023-35696-2)
Supplement: Supplementary file 1 — Supplementary Tables. [file 41598_2023_35696_MOESM1_ESM.pdf]

Supplementary file

Title: Myopia Progression Risk Assessment Score (MPRAS) – A promising new tool for risk stratification

Authors: Manoj K. Manoharan, Swapnil Thakur, Rohit Dhakal, Satish K. Gupta, Jacinth J. Priscilla, Shashank K. Bhandary, Alok Srivastava, Srinivas Marmamula, Nitish Poigal and Pavan K. Verkicharla

Supplementary Table S1: Myopia risk protocol incorporating various risk factors categorized into low, intermediate, and high-risk in model-2.

**Myopia risk factors**	**Low-risk**	**Score**	**Intermediate-risk**	**Score**	**High-risk**	**Score**
Change in SER or AL (D or mm change/year)	>-0.50 or <0.10	1	-0.50 to -0.75 or ≥0.10 to <0.20	4	<-0.75 or ≥0.20	6
Age of presentation (years)	≥18	1	>13 to <18	2	≤13	3
SER (D) at presentation	≥-3.00	1	<-3.00 to >-6.00	2	≤ -6.00	3
Age of onset (years)	≥18	1	>13 to <18	2	≤13	3
Time spent outdoors (hours per day)	>2	1	1 to 2	2	<1	3
RPR at presentation (Hyperopic defocus (D))	<+0.25	1	≥0.25 to +0.50	2	>+0.50	3
Time spent on near work (hours per day)	≤7	1	>7 to 10	2	>10	3
Near esophoria (PD)	≤2	1	>2 to <6	2	≥6	3
Accommodative lag (D)	≤+0.50	1	+0.75 to +1.00	2	>+1.00	3
Myopic parents	0	1	1	2	2	3

Abbreviations: SER-Spherical equivalent refractive error, AL-Axial length, RPR-Relative peripheral refraction, D-Dioptre, mm-millimetre, PD-Prism dioptre, Change in SER – the refraction at current eye examination subtracted from the refraction (or spectacle prescription) value obtained 1-year ago during previous examination, Change in AL - the central axial length at current eye examination subtracted from the axial length value obtained 1-year ago during previous examination

Supplementary Table S2: Examples for myopic individuals with the weightage model-1

SL No	Myopia risk factors	Low risk	Score		Intermediate risk	Score		High risk	Score	
			**Eg.1: Low risk case**	**Eg.2: Risk case**		**Eg.1: Low risk case**	**Eg.2: Risk case**		**Eg.1: Low risk case**	**Eg.2: Risk case**
1	Change in SER or AL (D or mm change/year)	>-0.50 or <0.10	1	0	-0.50 to -0.75 or ≥0.10 to <0.20	0	0	<-0.75 or ≥0.20	0	3
2	Age at presentation (years)	≥18	1	0	>13 to <18	0	0	≤13	0	3
3	SER (D) at presentation	≥-3.00	1	1	<-3 to >-6	0	0	≤-6.00	0	0
4	Age of onset (years)	≥18	0	0	>13 to <18	2	0	≤13	0	3
5	Time spent outdoors (hours per day)	>2	1	0	1 to 2	0	2	<1	0	0
6	RPR at presentation (Hyperopic defocus (D))	<+0.25	1	0	≥+0.25 to +0.50	0	0	>+0.50	0	3
7	Time spent on near work (hours per day)	≤7	1	1	>7 to 10	0	0	>10	0	0
8	Near esophoria (PD)	≤2	1	1	>2 to <6	0	0	≥6	0	0
9	Accommodative lag (D)	≤+0.50	0	1	+0.75 to +1.00	2	0	>+1.00	0	0
10	Myopic parents	0	0	0	1	0	0	2	3	3
		Total	A=7	A=4		B=4	B=2		C=3	C=15
		A, B, and C are the total score for each level of risk<br>Final MPRAS model = (1xA) + (2xB) + (3xC)<br>Eg.1: Final MPRAS = 24 & Eg.2: Final MPRAS = 53								

Supplementary Table S3: The Youden’s J-index, sensitivity, specificity, AUC, and cut-off values of the model-1 and -2 comparisons with the clinical decisions of the clinicians at the ‘Myopia Centre’ for the random eye analysis. In this table, C 1, C 2, C 3, C 4, and C 5 represent the first, second, third, fourth, and fifth clinicians’ respectively.

	Youden's J-index	Sensitivity	Specificity	AUC	Cut-off
**Model 1**					
C 1	0.65	0.84	0.81	0.90 ± 0.03	41.50
C2	0.65	0.84	0.81	0.90 ± 0.03	41.50
C 3	0.64	0.78	0.87	0.90 ± 0.03	43.50
C 4	0.64	0.85	0.79	0.90 ± 0.03	41.50
C 5	0.63	0.83	0.80	0.89 ± 0.03	41.50
**Model 2**					
C 1	0.76	0.90	0.86	0.94 ± 0.02	41.75
C 2	0.76	0.90	0.86	0.94 ± 0.02	41.75
C 3	0.74	0.90	0.84	0.94 ± 0.02	41.75
C 4	0.75	0.91	0.84	0.94 ± 0.02	41.75
C 5	0.75	0.89	0.86	0.93 ± 0.02	41.75
